# In Vitro Comparison of Macrophage Polarization and Osteoblast Differentiation Potentials between Granules and Block Forms of Deproteinized Bovine Bone Mineral

**DOI:** 10.3390/ma13122682

**Published:** 2020-06-12

**Authors:** Masako Fujioka-Kobayashi, Simon D. Marjanowski, Michihide Kono, Hiroki Katagiri, Richard J. Miron, Benoit Schaller

**Affiliations:** 1 Department of Cranio-Maxillofacial Surgery, Inselspital, Bern University Hospital, University of Bern, 3010 Bern, Switzerland; simon.marjanowski@insel.ch (S.D.M.); mk-oms@tokyo-med.ac.jp (M.K.); katagiri@ngt.ndu.ac.jp (H.K.); benoit.schaller@insel.ch (B.S.); 2 Department of Oral and Maxillofacial Surgery, Tokyo Medical University, Tokyo 113-8510, Japan; 3 Advanced Research Center, School of Life Dentistry at Niigata, The Nippon Dental University, Niigata 951-8151, Japan; 4 Department of Periodontology, University of Bern, 3010 Bern, Switzerland; richard.miron@zmk.unibe.ch

**Keywords:** deproteinized bovine bone mineral, guided bone regeneration, GBR, biocompatibility, macrophage polarization, osteoblast differentiation

## Abstract

Deproteinized bovine bone mineral (DBBM) bone grafts are commonly utilized for guided bone regeneration (GBR) techniques in regenerative dentistry. It has been hypothesized that different forms (blocks versus particulates) might demonstrate the varying properties of cell behavior during the regenerative process. Therefore, the aim of the present study was to investigate DBBM granules and blocks for their effects on osteoblasts and macrophages (Mφs). DBBM granules and blocks were filled to the same size (φ6.4 mm in diameter × 2.0 mm in height) in cell culture wells and assessed for cell viability and cell differentiation of human osteoblast-like Saos-2 cells, and Mφs derived from human monocyte THP-1 cells. The two groups were first characterized by micro-CT analysis, which demonstrated that DBBM granules had a two-fold greater material volume and a four-fold larger surface area than the blocks. DBBM blocks showed superior viability for both osteoblasts and Mφs. Osteoblast experiments were then utilized to better characterize the influence of DBBM shapes/forms on osteoblast differentiation. Alkaline phosphatase (ALP) staining on the undecalcified frozen sections was observed throughout the DBBM granule surface, yet this staining was only observed on the upper portion of the DBBM blocks. Furthermore, DBBM blocks showed M1-Mφ polarization trends with higher IL-1 and IL-6 mRNA expression in Mφs, while the conditioned media from Mφs cultured on DBBM granules promoted osteoblast differentiation with higher mRNA levels of Runx 2, ALP and osteocalcin. In conclusion, the DBBM granules showed more regenerative effects, lower M1 marker expression, and higher osteoblast differentiation potential when compared with the blocks, which might be related to the larger material volume, higher surface area and greater ability for the cells to penetrate through the scaffold.

## 1. Introduction

Guided bone regeneration (GBR) is a surgical technique using bone grafting materials to support bone formation in osseous defects [1,2]. Autologous bone is considered the gold standard material, owing to its osteoinductivity (bone-forming growth factors) and osteoconductivity (support of three-dimensional bone ingrowth within the scaffold), as well as providing osteogenic cells. Other available alternatives include allografts from freeze-dried human donors, xenografts from animal sources, and synthetic materials, all of which have been clinically utilized without the disadvantages of autografts, including additional surgical procedures, donor site morbidity, and extra surgical expenses [3,4]. Xenogenic bone substitutes, deproteinized bovine bone mineral (DBBM) granules have widely been utilized in regenerative dentistry for the management of periodontal bone defects, extraction socket preservation, alveolar ridge augmentation, and sinus floor elevation without disease transmission [5]. Despite very limited material resorption, over the years, DBBM granules have provided excellent osteoconductivity and new bone stability without adverse effects [6,7]. In addition, more recently, a block form of DBBM has been brought to the market for cases requiring a ridge contour increase or volume stability of the augmented region [8].

Nevertheless, the efficacy of DBBM blocks on bone regeneration remains controversial [9]. Recently, Benic et al., compared DBBM granules and blocks in a peri-implant defect GBR model in dogs [10,11]. The DBBM blocks resulted in superior outcomes with regard to the volume of the augmented ridge, when compared with empty controls by cone beam computed tomography (CBCT) analysis and histomorphometry, after 4 months of healing [10,11]. However, minimal new bone formation and graft material integration were observed in DBBM blocks, when compared with granules [10]. Similarly, Schwarz et al., reported relatively poor osteoconductivity of DBBM blocks, when compared with DBBM granules following lateral ridge augmentation in a dog model for 8 weeks [12]. Sawada et al., observed limited osteoconductivity of DBBM blocks in the peripheral region near the bone defect edge in a dog calvarial defect model, 12 months after implantation [13].

Interestingly, recent studies have shown that the shape of the bone substitutes is related to the tissue reaction and the bone-forming potential. Ghanaati et al., showed that changes in the size, shape and porosity of β-tricalcium phosphate (β-TCP) substitutes influenced biomaterial integration, multinucleated giant cell (MNGC) formation, and angiogenesis in a rat subcutaneous implant model [14]. Tanuma et al., reported that smaller octacalcium phosphate (OCP) granules with atelocollagen enhanced the capability to regenerate new bone, when compared with the larger OCP sizes in rat critical-sized calvarial defects [15]. Furthermore, Tamimi et al., demonstrated that different shapes of dicalcium phosphate dihydrate affected bone formation patterns and the osseointegration of dental implants, by using a 3D-printer fabrication method in a rabbit onlay model [16]. However, differences regarding the osteogenic potential and macrophage (Mφ) polarization potential between DBBM granules and blocks have not yet been clarified in vitro.

It was hypothesized that the different shapes/forms of DBBM might have different effects on cell behavior during the regenerative process. Therefore, the aim of the present study was to investigate DBBM granules and blocks for their effects on osteoblasts and Mφs. The DBBM granules and blocks were characterized by their density, volume and surface area by micro-CT, and further assessed for their effects on cell viability and cell differentiation in human osteoblast-like Saos-2 cells, and Mφs derived from human monocyte THP-1 cells for macrophage polarization.

## 2. Materials and Methods

### 2.1. Materials

Two types of DBBM materials (Bio-Oss^®^ granules, 0.25–1.0 mm and Bio-Oss^®^ Spongiosa blocks, 1 cm × 1 cm × 2 cm, Geistlich Pharma AG, Wolhusen, Switzerland) were tested in the present study. Both DBBM materials were aseptically prepared into the same size (φ6.4 mm in diameter × 2.0 mm in height), in 96-well plates or Transwell^®^ inserts (Sigma, St. Louis, MO, USA). The same size of the defect filling, instead of the same weight of the materials, was utilized for material comparison. This is usually the same concept as that in preclinical studies [10,11], especially when using the different forms of biomaterials, and allowed for a greater similarity with the clinical reality of the bone augmentation procedure.

### 2.2. Micro-CT Analysis

The materials were subjected to micro-CT scanning using a desktop Cone-Beam scanner (microCT 40, Scanco Medical AG, Brüttisellen, Switzerland). The X-ray source was set at 70 kVp, with 114 μA, at an isotropic voxel size of 6 µm. The obtained DICOM images were then segmented, and the material density, volume and surface area were measured using 3D structural analysis software (Amira 6.1, Visualization Sciences Group, Duesseldorf, Germany).

### 2.3. Cell Culture

THP-1 human monocytes (ATCC, Manassas, VA, USA) and human osteoblast-like Saos-2 cells (Sigma) were utilized. THP-1 cells were cultured in RPMI 1640 (Gibco, Life Technologies, Carlsbad, CA, USA), supplemented with 10% fetal bovine serum (FBS, ATCC), 100 units/mL penicillin, and 100 µg/mL streptomycin (Sigma) at 37 °C in a 5% CO_2_ atmosphere. THP-1 monocytes were treated with 100 ng/mL phorbol 12-myristate 13-acetate (PMA, Sigma) for 24 h, in order to facilitate their differentiation into Mφs (M0_Mφs). Saos-2 cells were grown in McCoy’s 5A (Gibco) with 10% FBS (Gibco), and antibiotic-antimycotic (Gibco; 100 units/mL penicillin, 100 µg/mL streptomycin and 250 ng/mL amphotericin B).

### 2.4. Cell Viability

Either THP-1-derived M0_Mφs or Saos-2 cells were cultured on control plastic culture plates, DBBM granules, or blocks at a density of 5.0 × 10^4^ cells per well in 96-well culture plates. Cell viability assays (CellTiter-Glo^®^, Promega, Madison, WI, USA) were performed on days 1 and 3. Briefly, a volume of CellTiter-Glo reagent equal to that of the culture medium was added, followed by mixing with an orbital shaker for 2 min; cells were then incubated at room temperature for 10 min. The luminescence signals in the incubated solution transferred into opaque 96-well plates were measured by a luminescence plate reader (TECAN Infinite 200, Tecan Group Ltd., Männedorf, Switzerland).

### 2.5. Real-Time PCR Analysis

Total RNA was extracted using a ReliaPrep™ RNA Cell Miniprep System (Promega) and quantified by a Nanodrop 2000 (Thermo, Wilmington, DE, USA). For RT-PCR, 0.5 μg of total RNA from each sample was used for cDNA synthesis using the GoScript™ Reverse Transcriptase system (Promega). Real-time PCR was performed on a real-time PCR system (7500 Fast Machine, Applied Biosystems, Thermo) using GoTaq^®^ qPCR Master Mix (Promega). The comparative Ct (∆∆Ct) method was used to calculate the gene expression levels after normalization, according to the expression of glyceraldehyde 3-phosphate dehydrogenase (GAPDH). The data show the relative values to the mRNA levels when cultured on control tissue culture plastic. The primers and their sequences are shown in Table 1.

### 2.6. Undecalcified Frozen Section Preparation and Staining

THP-1-derived M0 Mφs and Saos-2 cells were seeded on either DBBM granules or blocks at a density of 1.0 × 10^5^ cells per insert, in 6.5 mm Transwell inserts with 0.4-μm pore polycarbonate membranes in 24-well plates. After 3 days for THP-1 cells, and after 14 days for Saos-2 cells, the cells were fixed in 4% paraformaldehyde for 15 min, and immediately frozen in Super Cryoembedding Medium (SCEM; Section-Lab, Hiroshima, Japan) in the stainless steel container as previously described [17]. Sections (10 µm) were cut by a cryomicrotome (Microm HM550, Thermo) with an adhesive film (Cryofilm Type 2C (16UF), Section-Lab) using Kawamoto’s film method [18].

Alkaline phosphatase (ALP) staining was performed using the leukocyte alkaline phosphatase kit (procedure No. 86, Sigma, St. Louis, MO, USA) as reported previously [19]. Briefly, the sections were further fixed in a citrate-acetone-formaldehyde fixative solution for 5 min, and then incubated in the alkaline dye mixture solution consisting of 1 mL of sodium nitrite solution and 1 mL of fast red violet alkaline solution, in 45 mL of distilled water and 1 mL of Naphthol AS-Bl alkaline solution, for 15 min, protected from light, at 37 °C. Immunocytochemistry was performed as previously described [17]. Briefly, after permeabilization with 0.2% Triton X-100 in Phosphate-buffered saline (PBS) for 10 min, the sections were blocked in 2% goat serum/PBS (Dako, Jena, Germany) for 1 h. Subsequently, the specimens were incubated overnight at 4 °C with primary antibodies against CD68 (Abcam, London, UK, dilution 1:100), CD86 (Santa Cruz, CA, USA, dilution 1:50), or CD206 (Santa Cruz, dilution 1:50). The linked antibodies were detected by horseradish peroxidase-conjugated secondary antibodies (ScyTek Laboratories, Logan, UT, USA) for 1 h at room temperature, and 3,3’-Diaminobenzidine (ScyTek Laboratories). Counterstaining was performed with hematoxylin. All images were captured with a digital microscope (VHX-6000, Keyence, Osaka, Japan).

### 2.7. Conditioned Media Experiment

To investigate the effects of Mφs stimulated by DBBM on osteoblasts, Saos-2 cells were cultured in 20% conditioned media, collected from the Mφs cultured on the DBBM materials for 3 days. For the controls, media extracts incubated without cells were used. Cell viability assays were performed on days 1 and 3, after the seeding of Saos-2 cells. Moreover, the mRNA levels of TGF-β, Runx 2, COL1a2, ALP, and OCN were evaluated by real-time PCR on days 3 and 14. ALP staining was performed on day 7. Furthermore, after stimulation for 14 days with conditioned media in osteogenic differentiation medium, growth media supplemented with 50 µg/mL ascorbic acid (Sigma) and 10 mM β-glycerophosphate (Sigma), cells were fixed in 95% ethanol for 15 min and stained with 0.2% alizarin red (alizarin red S, Sigma) in distilled water (pH 6.4) at room temperature for 1 h. The stained areas were measured by the counting tool of the digital microscope (Keyence).

### 2.8. Statistical Analysis

The cell experiments were performed in triplicate, with three independent experiments for each condition. The mean and standard error were analyzed for statistical significance using the unpaired t-test (* *p* values <0.05 were considered significant) with GraphPad Prism 8.0 software (GraphPad Software, Inc., La Jolla, CA, USA).

## 3. Results

### 3.1. Micro-CT Analysis

**Figure 1:**
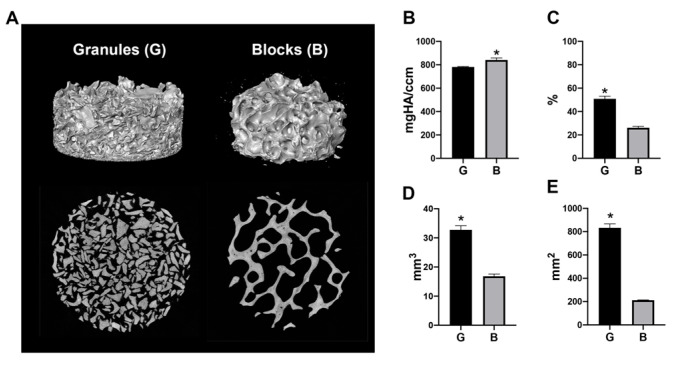
The material volume and surface area of deproteinized bovine bone mineral (DBBM) granules and blocks filled in a 6.4 mm diameter × 2 mm height cylinder. (**A**) Three-dimensional (3D) reconstructed images and 2D slice views of the materials. DBBM granules showed a higher fill density. (**B**) The material density (mgHA/ccm), (**C**) material volume in total volume (%), (**D**) material volume (mm^3^) and (**E**) material surface area (mm^2^) of DBBM granules (shown as “G”) and blocks (shown as “B”). (* Denotes significantly higher than the other group, *p* < 0.05.).

The same areas of DBBM granules and blocks were evaluated for their characteristics by micro-CT (Figure 1). A slightly higher material density was observed in DBBM blocks, when compared with the granules (Figure 1B). Interestingly, the quantitative results showed two-fold greater material volumes and a four-fold larger surface area in DBBM granules compared with the blocks, owing to their greater packing ability (Figure 1C–E).

### 3.2. The Direct Effects of DBBM Shapes on Osteoblast Differentiation

**Figure 2:**
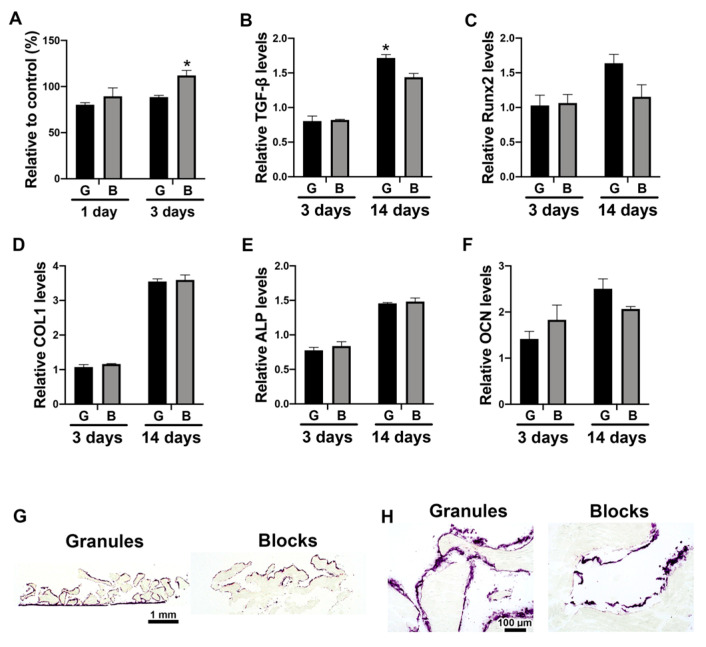
Osteoblast differentiation on DBBM granules or blocks. (**A**) Viability assay of Saos-2 cells cultured on DBBM granules (shown as “G”) or blocks (shown as “B”) at days 1 and 3. (* Denotes significantly higher than the other group, *p* < 0.05). (**B**–**F**) Real-time PCR of Saos-2 cells seeded on DBBM granules or blocks for genes encoding (**B**) TGF-β, (**C**) Runx 2, (**D**) COL1, (**E**) ALP, and (**F**) OCN at days 3 and 14 after cell seeding. (* Denotes significantly higher than the other group, *p* < 0.05). (**G**,**H**) ALP staining of undecalcified frozen sections of Saos-2 cells cultured on DBBM granules or blocks for 14 days. The violet-stained cells demonstrate ALP activity. (Original magnification (**G**) 50×, (**H**) 500×).

Saos-2 cells were cultured on either DBBM granules or blocks and investigated for their osteogenic potential. Very similar trends of osteoblast differentiation were observed between the two groups, except for significantly lower viability at day 3, and higher mRNA levels of TGF-β at day 14 in the cells cultured on DBBM granules, when compared with the blocks (Figure 2). Interestingly, a marked increase in ALP activity was observed surrounding DBBM granules, which was only observed on the upper portion of the DBBM blocks (Figure 2G,H).

### 3.3. The Effects of DBBM Shapes on Macrophage Polarization

**Figure 3:**
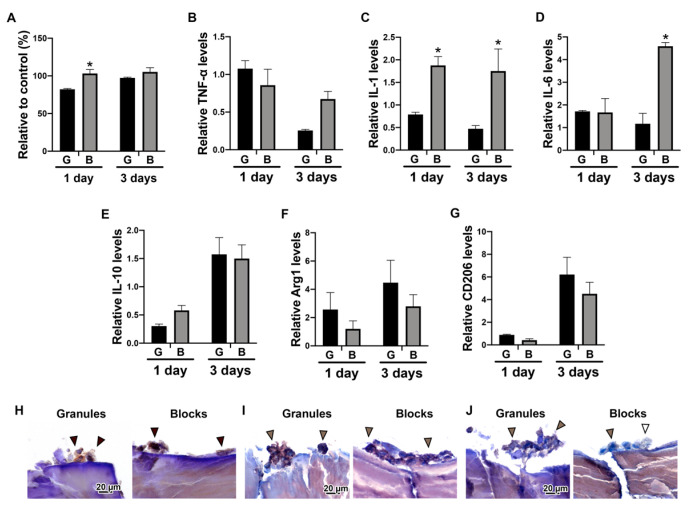
Macrophage polarization potential on DBBM granules or blocks. (**A**) Viability assay of THP-1-derived Mφs cultured on DBBM granules (shown as “G”) or blocks (shown as “B”) on days 1 and 3. (* Denotes significantly higher than the other group, *p* < 0.05). (**B**–**G**) Real-time PCR of Mφs seeded on DBBM granules or blocks for genes encoding (**B**) TNF-α, (**C**) IL-1, (**D**) IL-6, (**E**) IL-10, (**F**) Arg1, and (**G**) CD206 on days 1 and 3 after cell seeding. (* Denotes significantly higher than the other group, *p* < 0.05). (**H**–**J**) Immunochemical staining against (**H**) CD68, (**I**) CD86 and (**J**) CD206 on undecalcified frozen sections of Mφs cultured on DBBM granules or blocks for 3 days. (Original magnification 1000×).

The viability and expression levels of Mφ polarization were investigated when THP-1-derived Mφs were directly cultured on either DBBM granules or blocks (Figure 3). Higher viability was observed for Mφs cultured on DBBM blocks on day 1, when compared with the DBBM granules (Figure 3A). Furthermore, real-time PCR experiments showed higher mRNA levels of M1 markers, including IL-1 and IL-6, on DBBM blocks at day 3 (Figure 3C,D). However, there was no significant difference in M2 marker expression, including IL-10, Arg1 and CD206, between the two groups (Figure 3E–G). Immunochemical staining of the Mφs on DBBM materials revealed that CD68 and CD86 were expressed on both DBBM granules and blocks. Nevertheless, CD206 was only slightly positive on both groups (Figure 3H–J). The DBBM blocks tended to promote the M1 phenotype polarization by both real-time PCR and immunochemical staining, when compared with DBBM granules. However, both groups demonstrated no evidence that the Mφs were polarized toward the M2 phenotype.

### 3.4. The Paracrine Effects of Macrophage Conditioned Media Cultured on DBBM on Osteoblast Differentiation

**Figure 4:**
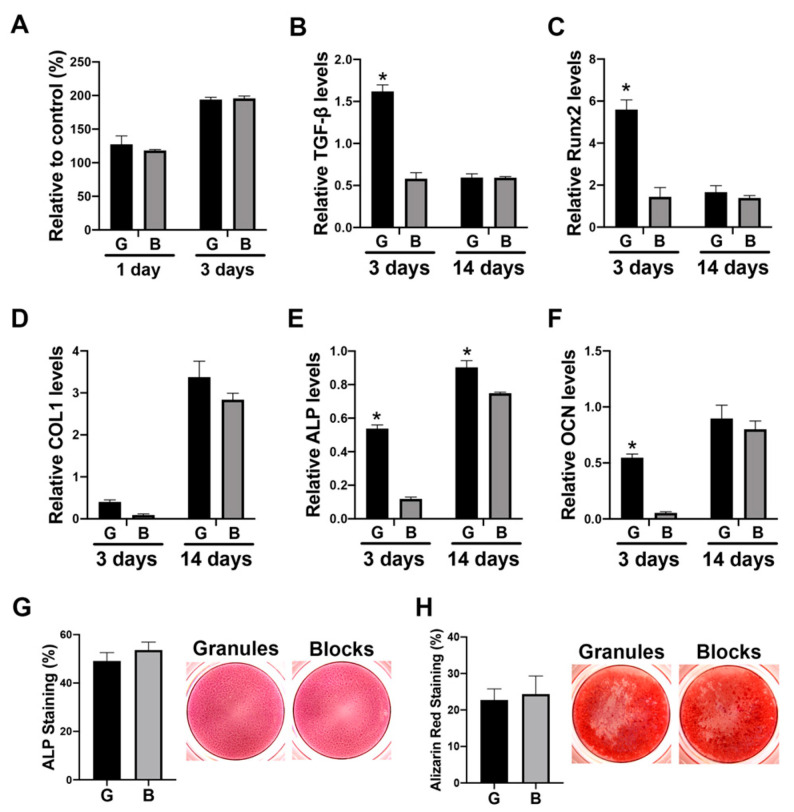
Osteoblast differentiation in conditioned media from Mφs cultured on either DBBM granules or blocks. (**A**) Viability assay of Saos-2 cells stimulated in conditioned media from Mφs cultured on DBBM granules (shown as “G”) or blocks (shown as “B”) on days 1 and 3. (**B**–**F**) Real-time PCR of Saos-2 cells seeded in conditioned media from Mφs cultured on DBBM granules or blocks for genes encoding (**B**) TGF-β, (**C**) Runx 2, (**D**) COL1, (**E**) ALP, and (**F**) OCN on days 3 and 14 after cell seeding. (* Denotes significantly higher than the other group, *p* < 0.05). (**G**) ALP staining of Saos-2 cells cultured in conditioned media for 7 days. (**H**) Alizarin red staining of Saos-2 cells cultured in conditioned media with osteoblast differentiation media for 14 days.

The paracrine effects of DBBM-mediated Mφs on osteoblasts were further investigated (Figure 4). The DBBM conditioned media did not influence cell viability on days 1 or 3 (Figure 4A). Real-time PCR showed that DBBM granule-conditioned media demonstrated higher levels of osteoblastic markers, including TGF-β, Runx 2, ALP, and OCN, especially on day 3 compared with the blocks (Figure 4B–F). No significant differences in either ALP or alizarin red staining were observed between the DBBM granules and blocks (Figure 4G,H).

## 4. Discussion

Commercially available bone substitutes are integrated into host tissues as foreign bodies with varying degrees of inflammation, and are typically resorbed and replaced by vital bone over time [20,21]. Multinucleated giant cells (MNGCs) are consistently detected around bone substitutes following bone augmentation [22,23]. Frequently, however, a material-associated foreign body reaction is ignored when the results of successful bone formation are reported. Foreign body reactions are mostly associated with macrophages, which engulf and digest cellular debris and foreign substances during phagocytosis. Recently, it was recognized that biomaterial-associated macrophages may be both pro-inflammatory (M1 Mφ) and tissue regenerative (M2 Mφ), and that the phenotype may change from M1 to M2 and vice versa [24]. Upon biomaterial implantation, the initial M1 response is responsible for recruiting inflammatory cells to the site of injury and instigating the ensuing foreign body reaction, and a subsequent transition to the M2 phenotype [25]. Such Mφ plasticity is essential for rapid inflammation reduction, and results in greater vascularization, tissue remodeling and wound healing [26]. However, the difference in the Mφ behavior between DBBM granules and blocks had not yet been clarified. Therefore, the effects of DBBM materials on Mφ polarization potential were investigated in the present study.

Interestingly, the Mφ culture experiment on DBBM materials showed higher M1 markers expression, including IL-1 and IL-6, on the DBBM blocks than on the granules on day 3, but there was no significant difference in M2 marker expression, including IL-10, Arg1 and CD206, between the two material groups (Figure 3B–G). Previously, Shi et al., reported that DBBM granules promoted Mφ fusion and polarization toward an M2 phenotype with higher expression levels of CD163 and CD206, when compared with control tissue culture plastic in the murine macrophage cell line RAW 264.7 [27]. In line with Shi et al., the present study showed relatively lower mRNA levels of M1 markers, including TNF-α, IL-1, and IL-6, as well as higher mRNA levels of M2 markers, including IL-10, Arg1 and CD206 on day 3 on DBBM materials, compared with control tissue culture plastic. However, the relative mRNA levels to control tissue culture plastic are presented in the present study, in order to focus on the comparison of the results between granules and blocks.

In line with the real-time PCR results, immunochemical staining of Mφs on DBBM materials, on undecalcified frozen sections, demonstrated positive expression of CD68 and CD86, but also slightly positive expression of CD206 on both materials (Figure 3H–J). This staining approach using undecalcified sections is a new method, especially for in vitro bone substitute research. The properties of bone substitutes are difficult to test when using cell culture systems because of their mineral contents, which interfere with the observation of the cell behavior of the materials. Kawamoto’s film transfer procedure [18] was used in the present study to visualize cell localization and Mφ marker expression on DBBM materials at the protein level. Traditional methods, such as the paraffin embedding procedure, require decalcification and liquid handling, which destroys the fragile structure of cells cultured in vitro on materials. Moreover, Transwell^®^ inserts, whose membranes allow only liquid passage, successfully maintained granule stability during the liquid handling and the collection of samples in 24-well plates, as previously reported [17]. Different cell localization was clearly observed between DBBM granules and blocks by ALP staining of Saos-2 cells, showing a marked increase in ALP expression surrounding all DBBM granules. However, this expression was only observed on the upper portion of the DBBM blocks (Figure 2G,H). The undecalcified section preparation method might be a good alternative to investigate direct cell localization and phenotypes on hard tissues or materials in vitro.

Furthermore, osteoblast behavior on DBBM granules or blocks was investigated when cultured directly on materials, and in conditioned media from Mφs on DBBM materials. A similar trend for osteoblast differentiation was observed between the two different groups (granules versus blocks) in a direct culture system (Figure 2). However, the paracrine factors of DBBM-mediated Mφ on osteoblasts demonstrated a relatively different effect between the two materials. This resulted in higher mRNA levels of osteoblastic markers, including TGF-β, Runx 2, ALP, and OCN, especially on day 3 in Mφ-conditioned media on DBBM granules, when compared with the blocks (Figure 4B–F). Osteoblast culturing directly on bone substitutes is a common method to investigate biomaterials for their in vitro cytotoxicity [28]. However, Mφ-conditioned media experiments may better represent a more ideal environment when trying to evaluate the potential in vivo reaction. Nevertheless, it is quite challenging to assume the in vivo complex bone regeneration events only from the present in vitro results. Previously, Benic et al., compared DBBM granule and block materials in a peri-implant defect GBR model in dogs [10]. They reported that no significant difference was found in the new bone area between the two groups; however, superior osteoconductivity of DBBM granules was observed, compared with the blocks by histomorphometry [10]. It was suggested that the different osteoconductive potentials between DBBM granules and blocks were possibly due to differences in the macrostructure, with DBBM granules permitting superior blood vessel infiltration and new bone ingrowth [10]. The outcomes between DBBM granules and blocks on bone formative potential in vivo may be very similar to those in the present in vitro study, with the slightly greater osteogenic effects in DBBM granules especially at early time points.

The differences between cell reactions in Mφs and osteoblasts in the present study might also be related to material characteristics. The materials were made from the same animals (bovine) by similar deproteinization and heating methods at the same manufacturing facility. However, when considering the cell-attached area, the provided material volume/material surface in the created defect might be associated with cell reaction properties. There is also great interest in further evaluating the influence of the shapes of the other types of bone substitutes, including allografts and synthetic materials, on bone forming properties.

## 5. Conclusions

The effects of the DBBM form (granules or blocks) on osteoblast differentiation and Mφ differentiation were investigated. DBBM granules could be more densely filled into the same size defect when compared with DBBM blocks, with a higher volume and surface area. Small differences were observed in the effects of the two groups on osteoblast differentiation when cultured directly on the materials. However, interestingly, DBBM blocks showed higher M1-Mφ polarization trends with higher IL-1 and IL-6 mRNA levels. Furthermore, the conditioned media from Mφ cultured on DBBM granules promoted osteoblast differentiation with higher mRNA levels of Runx 2, ALP and osteocalcin. In summary, the DBBM granules showed more regenerative potential, lower M1 marker expression, and greater osteoblast differentiation when compared with the blocks, which might be related to the larger material volume and surface area.

## Figures and Tables

**Table 1:** Primers for quantitative RT-PCR.

Gene	Primer Sequence (5′–3′)	
	Forward	Reverse
TNF-α	CAGCCTCTTCTCCTTCCTGAT	GCCAGAGGGCTGATTAGAGA
IL-1	GGTTGAGTTTAAGCCAATCCA	TGCTGACCTAGGCTTGATGA
IL-6	GAAAGGAGACATGTAACAAGAGT	GATTTTCACCAGGCAAGTCT
IL-10	GAGGCTACGGCGCTGTCA	TCCACGGCCTTGCTCTTG
Arg-1	ACGGAAGAATCAGCCTGGTG	GTCCACGTCTCTCAAGCCAA
CD206	GGGTTGCTATCACTCTCTATGC	TTTCTTGTCTGTTGCCGTAGTT
TGF-β1	ACTACTACGCCAAGGAGGTCA	TGCTTGAACTTGTCATAGATTTCG
Runx 2	TCTTAGAACAAATTCTGCCCTTT	TGCTTTGGTCTTGAAATCACA
COL1a2	CCCAGCCAAGAACTGGTATAGG	GGCTGCCAGCATTGATAGTTTC
ALP	GACCTCCTCGGAAGACACTC	TGAAGGGCTTCTTGTCTGTG
OCN	AGCAAAGGTGCAGCCTTTGT	GCGCCTGGGTCTCTTCACT
GAPDH	AGCCACATCGCTCAGACA	GCCCAATACGACCAAATCC

TNF-α: tumor necrosis factor-α; IL: interleukin; Arg-1: arginase 1; TGF-β1: transforming growth factor-β; Runx 2: runt-related transcription factor 2; COL1: collagen 1; ALP: alkaline phosphatase; OCN: osteocalcin.

## References

[1] Retzepi M., Donos N. (2010). Guided Bone Regeneration: Biological principle and therapeutic applications. Clin. Oral Implant. Res..

[2] Zimmermann C., Gierloff M., Hedderich J., Açil Y., Wiltfang J., Terheyden H. (2011). Biocompatibility of bone graft substitutes: Effects on survival and proliferation of porcine multilineage stem cells in vitro. Folia Morphol..

[3] Giannoudis P.V., Dinopoulos H., Tsiridis E. (2005). Bone substitutes: An update. Injury.

[4] Schmitt C.M., Doering H., Schmidt T., Lutz R., Neukam F.W., Schlegel K.A. (2013). Histological results after maxillary sinus augmentation with Straumann^®^ BoneCeramic, Bio-Oss^®^, Puros^®^, and autologous bone. A randomized controlled clinical trial. Clin. Oral Implant. Res.

[5] Darby I. (2011). Periodontal materials. Aust. Dent. J..

[6] Zitzmann N.U., Naef R., Schärer P. (1997). Resorbable versus nonresorbable membranes in combination with Bio-Oss for guided bone regeneration. Int. J. Oral Maxillofac. Implant..

[7] Baldini N., De Sanctis M., Ferrari M. (2011). Deproteinized bovine bone in periodontal and implant surgery. Dent. Mater..

[8] Benic G.I., Hämmerle C.H. (2014). Horizontal bone augmentation by means of guided bone regeneration. Periodontology 2000.

[9] Hsu Y.T., Wang H.L. (2013). How to select replacement grafts for various periodontal and implant indications. Clin. Adv. Periodontics.

[10] Benic G.I., Thoma D.S., Muñoz F., Sanz Martin I., Jung R.E., Hämmerle C.H. (2016). Guided bone regeneration of peri-implant defects with particulated and block xenogenic bone substitutes. Clin. Oral Implant. Res..

[11] Benic G.I., Thoma D.S., Jung R.E., Sanz-Martín I., Unger S., Cantalapiedra A., Hämmerle C.H.F., Sanz I.M. (2017). Guided bone regeneration with particulate vs. block xenogenic bone substitutes: A pilot cone beam computed tomographic investigation. Clin. Oral Implant. Res..

[12] Schwarz F., Rothamel D., Herten M., Ferrari D., Sager M., Becker J. (2008). Lateral ridge augmentation using particulated or block bone substitutes biocoated with rhGDF-5 and rhBMP-2: An immunohistochemical study in dogs. Clin. Oral Implant. Res..

[13] Sawada K., Nakahara K., Haga-Tsujimura M., Iizuka T., Fujioka-Kobayashi M., Igarashi K., Saulacic N. (2018). Comparison of three block bone substitutes for bone regeneration: Long-term observation in the beagle dog. Odontology.

[14] Ghanaati S., Barbeck M., Orth C., Willershausen I., Thimm B.W., Hoffmann C., Rasic A., Sader R.A., Unger R.E., Peters F. (2010). Influence of β-tricalcium phosphate granule size and morphology on tissue reaction in vivo. Acta Biomater..

[15] Tanuma Y., Anada T., Honda Y., Kawai T., Kamakura S., Echigo S., Suzuki O. (2012). Granule Size–Dependent Bone Regenerative Capacity of Octacalcium Phosphate in Collagen Matrix. Tissue Eng. Part A.

[16] Tamimi F., Torres J., Al-Abedalla K., Lopez-Cabarcos E., Alkhraisat M.H., Bassett D.C., Gbureck U., Barralet J.E. (2014). Osseointegration of dental implants in 3D-printed synthetic onlay grafts customized according to bone metabolic activity in recipient site. Biomaterials.

[17] Fujioka-Kobayashi M., Ülgür I.I., Katagiri H., Vuignier S., Schaller B. (2020). In vitro observation of macrophage polarization and gingival fibroblast behavior on three-dimensional xenogeneic collagen matrixes. J. Biomed. Mater. Res. Part A.

[18] Kawamoto T., Kawamoto K. (2014). Preparation of thin frozen sections from nonfixed and undecalcified hard tissues using Kawamot’s film method (2012). Skeletal Development and Repair.

[19] Fujioka-Kobayashi M., Sawada K., Kobayashi E., Schaller B., Zhang Y., Miron R.J. (2017). Osteogenic potential of rhBMP9 combined with a bovine-derived natural bone mineral scaffold compared to rhBMP2. Clin. Oral Implant. Res..

[20] Galindo-Moreno P., Hernández-Cortés P., Mesa F., Carranza N., Juodzbalys G., Aguilar M., O′Valle F. (2013). Slow resorption of anorganic bovine bone by osteoclasts in maxillary sinus augmentation. Clin. Implant Dent. Relat. Res..

[21] Piattelli M., Favero G.A., Scarano A., Orsini G., Piattelli A. (1999). Bone reactions to anorganic bovine bone (Bio-Oss) used in sinus augmentation procedures: A histologic long-term report of 20 cases in humans. Int. J. Oral Maxillofac. Implant..

[22] Miron R.J., Zohdi H., Fujioka-Kobayashi M., Bosshardt D.D. (2016). Giant cells around bone biomaterials: Osteoclasts or multi-nucleated giant cells?. Acta Biomater..

[23] Barbeck M., Booms P., Unger R., Hoffmann V., Sader R., Kirkpatrick C.J., Ghanaati S. (2017). Multinucleated giant cells in the implant bed of bone substitutes are foreign body giant cells—New insights into the material-mediated healing process. J. Biomed. Mater. Res. Part A.

[24] Miron R.J., Bosshardt D.D. (2016). OsteoMacs: Key players around bone biomaterials. Biomaterials.

[25] Sridharan R., Cameron A.R., Kelly D.J., Kearney C.J., O′Brien F.J. (2015). Biomaterial based modulation of macrophage polarization: A review and suggested design principles. Mater. Today.

[26] Spiller K.L., Nassiri S., Witherel C.E., Anfang R.R., Ng J., Nakazawa K.R., Yu T., Vunjak-Novakovic G. (2015). Sequential delivery of immunomodulatory cytokines to facilitate the M1-to-M2 transition of macrophages and enhance vascularization of bone scaffolds. Biomaterials.

[27] Shi M., Wang C., Wang Y., Tang C., Miron R.J., Zhang Y. (2018). Deproteinized bovine bone matrix induces osteoblast differentiation via macrophage polarization. J. Biomed. Mater. Res. Part A.

[28] (2009). ISO: 2009 Biological Evaluation of Medical Devices—Part 5: Tests for In Vitro Cytotoxicity. Int. Organ. Stand. Geneva.

